# Supplementary material for: Profiling the interplay and coevolution of Microcystis aeruginosa and cyanosiphophage Mic1
Source: Microbiol Spectr. 2024 May 2;12(6):e00298-24. doi: 10.1128/spectrum.00298-24 (PMC11237433; doi:10.1128/spectrum.00298-24)
Supplement: Supplemental figures and tables — Fig. S1-S6; Tables S2-S6. [file spectrum.00298-24-s0001.pdf]

Supplementary information

Supplementary figures and figure legends

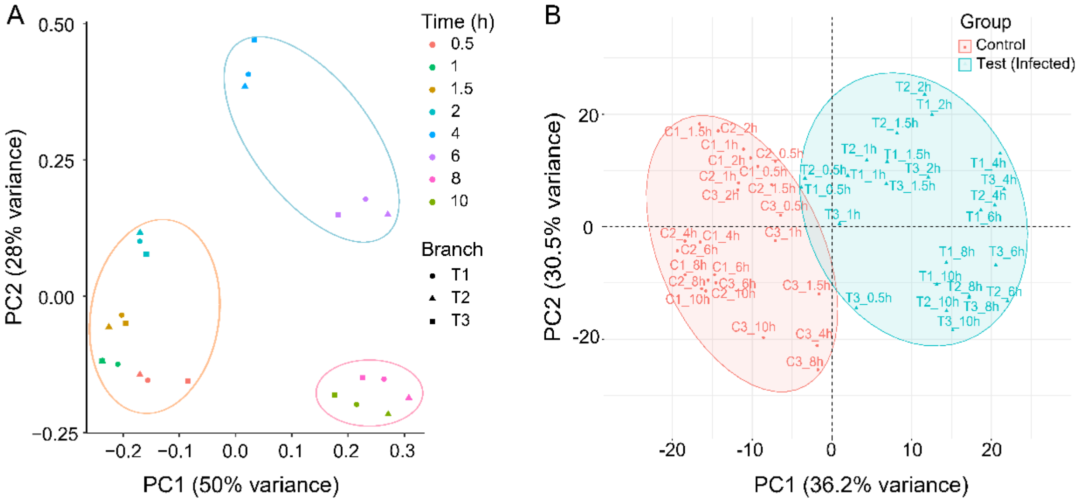

FIG S1 Principal component analysis (PCA) for clustering gene expression data, via Hotelling's 95% confidence ellipses. (A) Mic1 samples could be classified into three groups corresponding to the early, middle and late phase in the lytic cycle. Colors represent different infection time points, whereas shapes indicate three individual infection experiments. (B) PCA on the top 500 genes of host *Microcystis* from control (red) and infected (blue) cells, which shows a distinct clustering. Control: control cells; Test: infected cells.

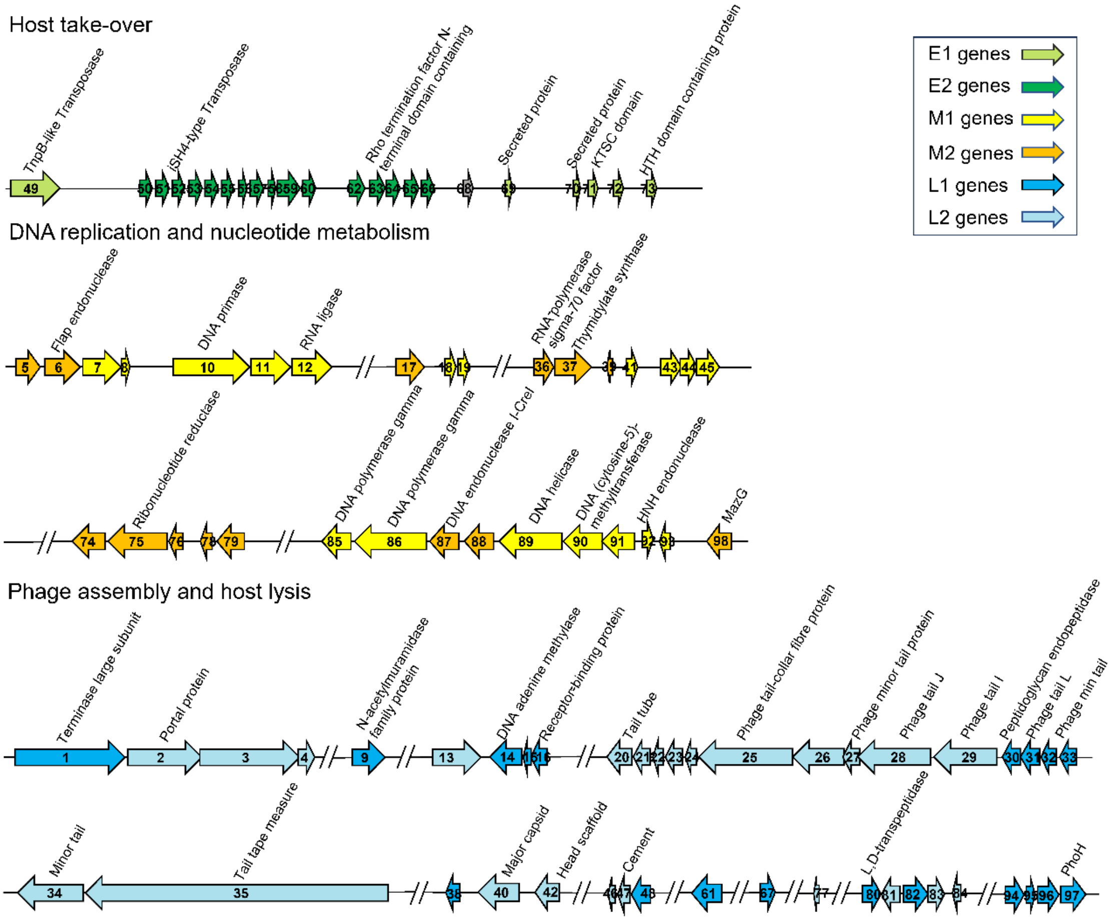

FIG S2 Presentation of 98 Mic1 genes according to the temporal expression patterns. The arrows indicate the transcription direction. The annotations of genes that contribute to the definite function of each temporal expression pattern are indicated.

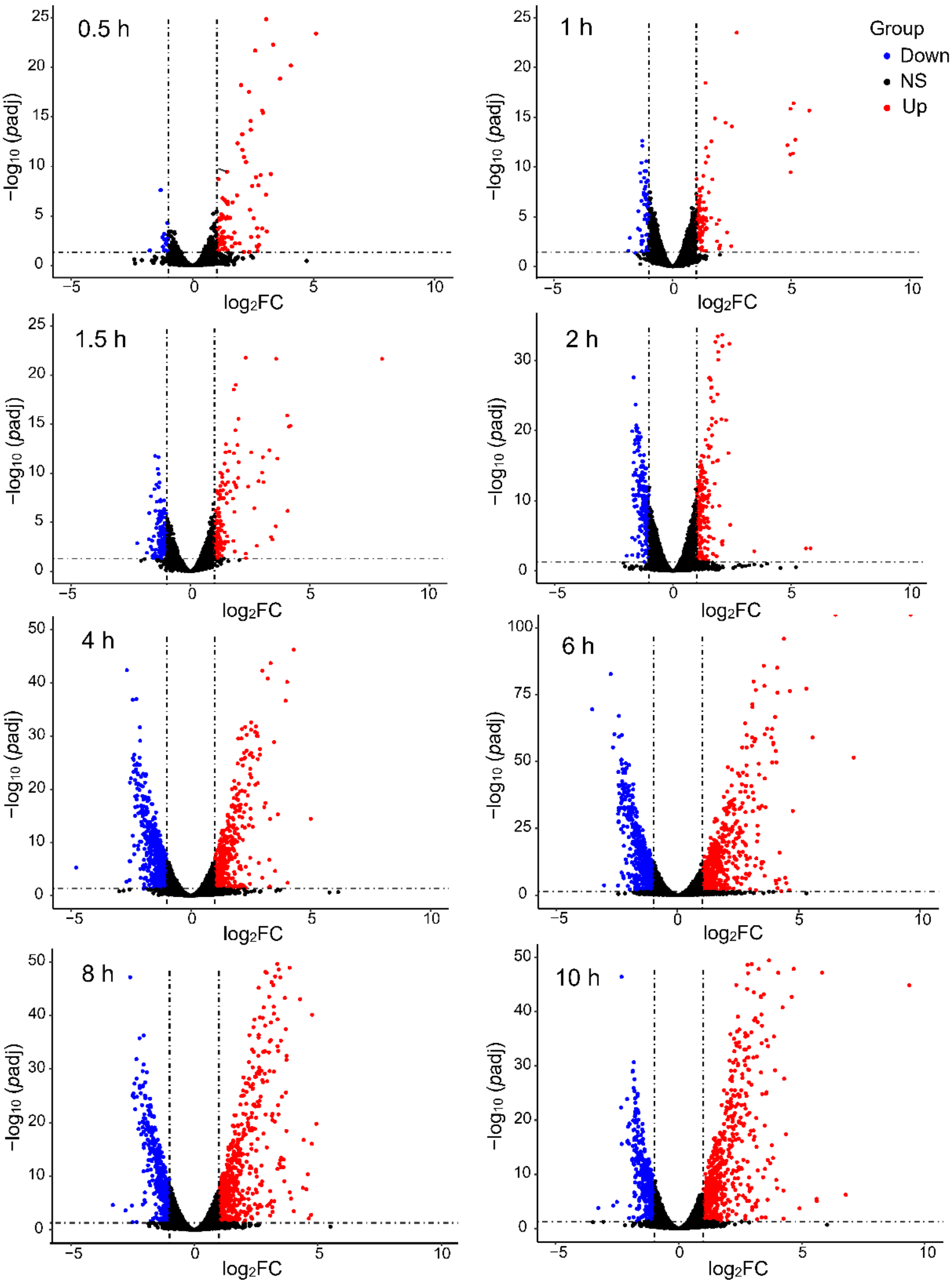

FIG S3 Volcano plots of the host DEGs at each time point. The $\log_2$ scale shows the fold change of genes, whereas the $\log_{10}$ scale represents the significance. Down: downregulated host genes; NS: not significant changed genes; Up: upregulated host genes.

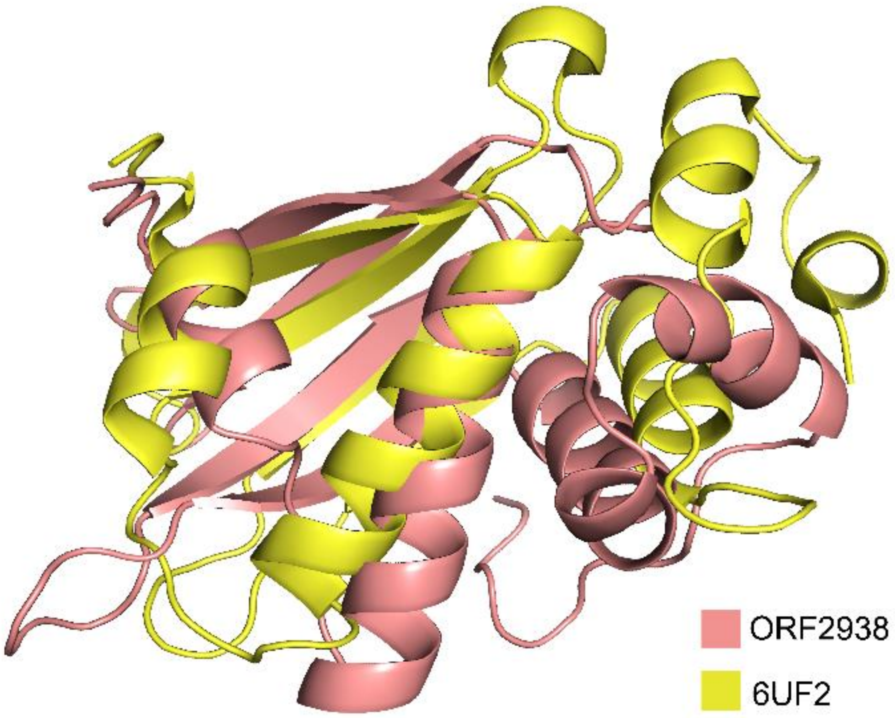

FIG S4 Structural superposition of *Microcystis* ORF2938 (salmon, predicated by Alphafold2) against the biofilm-related protein Se0862 from *Synechococcus elongatus* PCC 7942 (yellow, PDB: 6UF2).

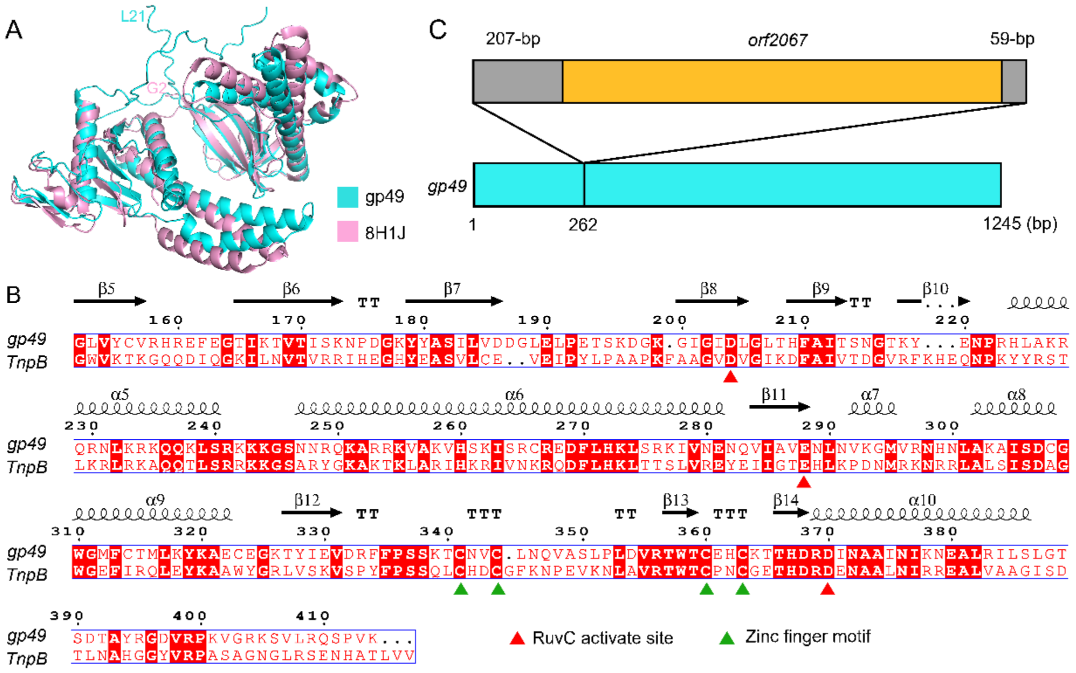

FIG S5 Analyses of Mic1 E1 gene *gp49*. (A) Structural superposition of the Mic1 gp49 (cyan, predicated by Alphafold2) against the RNA-guided DNA endonuclease TnpB from *Deinococcus radiodurans* R1 (pink, PDB: 8H1J). (B) Primary sequence alignment of the gp49 against TnpB. The key residues of zinc finger motif and RuvC activate site are indicated with green and red triangles, respectively. (C) A diagram of Mic1 *gp49* inserted with the host gene *orf2067*. Cyan: Mic1 *gp49*; yellow: *Microcystis orf2067*; gray: the upstream and downstream region of *orf2067*.

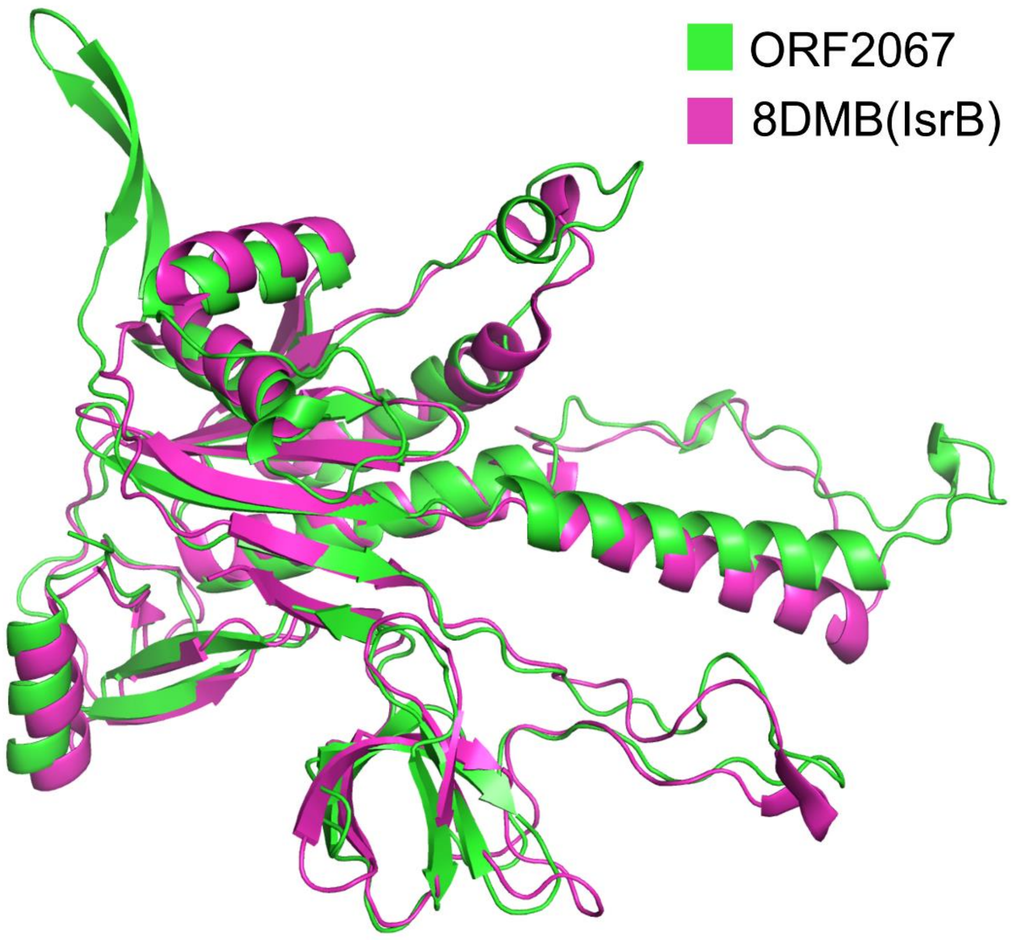

FIG S6 Structural comparison of *Microcystis* ORF2067 (magenta, predicated by Alphafold2) against *Desulfovirgula thermocuniculi* IsrB (green, PDB: 8DMB).

Supplementary tables

TABLE S2 Summary of the host response gene number after infection.

Time (h)	0.5	1	1.5	2	4	6	8	10
Up-regulated genes	109	113	163	241	410	560	588	583
Down-regulated genes	17	71	164	220	476	513	446	384

TABLE S3 Transcriptional level of a polyketide synthase pathway in the *Microcystis* genome after Mic1 infection.

Locus tag	Strand	Annotation	Log_2 FoldChange							
			**0.5 h**	**1 h**	**1.5 h**	**2 h**	**4 h**	**6 h**	**8 h**	**10 h**
orf2639	+	Transcriptional regulator	2.4612084	/	/	/	-1.63345	-1.04477	/	1.473188
orf2640	-	Hypothetical protein VL20_5146	6.4102847	5.760095	4.118665	2.086657	-2.36636	-1.2546	/	2.363854
orf2641	+	Polyketide synthase PksJ	5.5519478	4.958536	2.315594	/	-2.53106	-1.60033	/	2.872712
orf2642	+	Hypothetical protein	5.8659139	4.976635	2.669211	/	-2.19939	-1.68823	-1.72036	2.718858
orf2643	+	Phthiocerol/phenolphthiocerol polyketide synthase type I PpsB	6.1315641	5.076128	2.774814	/	-1.93521	-1.42861	-1.40167	2.546412
orf2644	+	Phenolphthiocerol synthesis polyketide synthase type I Pks15/1	4.2143615	4.194029	1.583222	/	-1.6985	-1.36727	-1.10605	2.13411
orf2645	+	Peptide synthetase	4.9906953	4.96781	3.370554	/	-1.32123	/	/	2.237928
orf2646	-	Multidrug resistance protein MdtC	4.4218152	4.516152	3.026797	/	/	1.207171	/	2.281126
orf2647	-	Solvent efflux pump periplasmic linker SrpA	2.5826113	2.702131	1.53083	/	/	/	/	1.953742
orf2648	-	VPDSG-CTERM motif (stress sensor)	5.8360923	5.209478	4.066525	1.042213	-1.83363	-1.59634	-1.65425	1.78915
orf2649	-	PEP-CTERM sorting domain-containing protein	5.3130824	5.166991	4.184787	2.426615	/	-1.07259	/	1.910546
orf2650	-	VPDSG-CTERM motif	5.0948386	4.977906	4.05174	1.836686	-1.93539	-1.46646	/	1.515961
orf2651	-	Dehydrogenase	4.0568476	4.604382	3.307606	/	-1.7753	-1.26227	-1.23494	2.398548
orf2652	-	Putative MFS-type transporter YcaD	2.8082371	3.475419	2.418648	/	-1.10678	-1.01994	/	2.195439
orf2653	-	Polyketide biosynthesis protein PksE	3.7209192	4.277037	2.997778	/	-1.07291	/	/	2.102777
orf2654	-	3-hydroxyacyl-[acyl-carrier-protein] dehydratase FabA	4.9043982	4.83869	3.571569	1.689592	-1.87002	-1.4065	-1.07553	2.139205
orf2655	-	Polyketide synthase PksN	5.2274829	5.09874	3.641738	/	-1.46475	-1.20346	-1.13286	2.445249
orf2656	-	Polyketide synthase modules and related proteins	4.4503857	4.411876	3.054075	/	-1.77759	/	-1.16285	2.398233
orf2657	-	Phenolphthiocerol synthesis polyketide synthase type I Pks15/1	4.2318081	4.171043	2.852323	/	-1.28183	/	/	2.402555

TABLE S4 Transcriptional level of a type III-B CRISPR pathway in the *Microcystis* genome after Mic1 infection.

Locus tag	Strand	Annotation	Log_2 FoldChange							
			0.5 h	1 h	1.5 h	2 h	4 h	6 h	8 h	10 h
orf1109	-	Cas_Csm6	/	/	/	1.30323	/	3.301023	4.283178	4.280763
orf1110	-	Type III-B CRISPR module RAMP protein Cmr6	/	/	/	/	1.372048	3.541163	4.530266	4.295065
orf1111	-	CRISPR-associated protein Cmr5	/	1.197197	/	1.474082	1.336261	3.615945	4.767138	4.59428
orf1112	-	Uma2 family endonuclease	/	/	1.114649	1.111276	1.416458	4.009954	5.256806	5.262678
orf1113	-	Type III-B CRISPR module RAMP protein Cmr4	/	/	/	1.290125	1.248191	4.106955	5.095018	5.106959
note=CRISPR with 2 repeat units										
orf1114	-	CRISPR-associated protein Cmr3	/	/	/	1.260325	/	2.896936	3.923709	3.66849
orf1115	-	Type III-B CRISPR-associated protein Cas10/Cmr2	1.00252	/	/	/	/	/	/	/
orf1116	-	WYL domain-containing protein	/	/	/	/	/	/	/	1.120162
note=CRISPR with 7 repeat units										

TABLE S5 KEGG enrichment analysis of the *Microcystis* downregulated protein-coding genes after Mic1 infection.

**Locus tag**	**Gene name**	**Annotation**	**Log$_2$ FoldChange**							
			**0.5 h**	**1 h**	**1.5 h**	**2 h**	**4 h**	**6 h**	**8 h**	**10 h**
**Photosynthesis pathway**										
orf141	*psbJ*	Photosystem II reaction center protein J	/	/	/	/	/	-1.201243	-2.087642	/
orf143	*psbF*	Cytochrome b559 subunit beta	/	/	/	/	-1.250366	-1.569605	-1.40205	-1.138037
orf144	*psbE*	Cytochrome b559 subunit alpha	/	/	/	/	-1.315481	-1.519895	-1.573858	-1.053689
orf330	*psaB*	Phosphorylase	/	/	/	/	-1.925144	-1.81953	-1.882642	-1.388888
orf331	*psaA*	Photosystem I core protein PsaA	/	/	/	-1.304793	-2.34749	-2.317547	-2.209604	-1.733649
orf345	*psaJ*	Photosystem I subunit IX	/	/	/	-1.178315	-2.190563	-2.218158	-2.258534	-1.844846
orf346	*psaF*	Photosystem I reaction center subunit III	/	/	/	-1.051194	-2.072846	-2.189871	-2.094665	-1.886535
orf377	*psbO*	Photosystem II manganese-stabilizing polypeptide	/	/	/	/	-1.455193	-1.420518	-1.051832	/
orf475	*psaK*	Photosystem I reaction center subunit PsaK	/	/	-1.285305	-1.524465	-2.133878	-2.427376	-2.219605	-1.869541
orf489	*cpcC*	Photosystem I reaction center subunit XII	/	/	-1.095086	-1.523684	-2.399969	-2.410539	-2.342115	-2.008888
orf490	*cpcC*	Photosystem I reaction center subunit XII	/	/	-1.053396	-1.510809	-2.284716	-2.450062	-2.345996	-2.000975
orf491	*cpcA*	Phycocyanin subunit alpha	/	/	-1.181315	-1.607822	-2.502661	-2.42828	-2.498428	-2.078517
orf492	*cpcB*	Phycocyanin subunit beta	/	/	-1.211637	-1.646318	-2.542182	-2.339128	-2.432239	-2.101509
orf555	*psbZ*	Photosystem II reaction center protein Z	/	/	-1.281835	-1.658332	-2.050432	-1.978609	-1.783488	-1.69584
orf569	*psaK*	Photosystem I reaction center subunit PsaK	/	/	/	/	-1.237088	-1.185189	-1.425413	-1.107851
orf594	*psaE*	Photosystem I reaction center subunit IV	/	/	-1.100902	-1.172105	-2.274811	-2.161566	-1.658397	-1.866168
orf661	*psaI*	Photosystem I reaction center subunit VIII	/	/	/	-1.468384	-2.554494	-2.317852	-2.001605	-1.204219
orf662	*psaL*	Photosystem I reaction center protein subunit XI	/	/	/	-1.027162	-2.382945	-2.292118	-2.039346	-1.633021
orf760	*petH*	Ferredoxin--NADP(+) reductase	/	/	/	/	-1.69618	-1.849362	-1.650852	-1.194849
orf1273	*apcE*	Photosystem I reaction center subunit X	/	/	/	-1.306494	-2.126029	-2.266192	-2.000028	-1.760948
orf1322	*apcD*	Allophycocyanin subunit alpha	/	/	/	-1.133682	-1.9737	-1.902514	-1.816066	-1.760642
orf1402	*ATPF1B, atpD*	ATP synthase subunit beta	/	/	/	-1.259526	-1.859781	-2.045115	-2.025824	-1.671682
orf1403	*ATPF1E, atpC*	ATP synthase epsilon chain	/	/	-1.019071	-1.265868	-1.879405	-1.999593	-1.650046	-1.492712
orf1433	*cpcG*	Phycobilisome rod-core linker polypeptide CpcG	/	/	-1.148142	-1.613261	-2.421204	-2.210494	-2.122146	-1.828206

orf1599	*psbV*	Cytochrome c-550	/	/	/	-1.175374	-2.19253	-2.008311	-1.762699	-1.446373
orf1863	*cpcE*		/	-1.139688	/	/	/	/	-1.106831	/
orf2052	*atpI*	ATP synthase protein I	/	/	/	/	-1.533646	-1.74822	-1.58791	-1.195098
orf2053	*ATPF0A, atpB*	AtpB	/	/	/	/	-1.496058	-1.67446	-1.660346	-1.270855
orf2054	*ATPF0C, atpE*	ATP synthase F0 subunit C	/	/	/	-1.03497	-1.413663	-1.793516	-2.041615	-1.421271
orf2055	*ATPF0B, atpF*	F0F1 ATP synthase subunit B'	/	/	-1.269863	-1.508116	-1.720013	-1.840708	-1.617089	-1.458756
orf2056	*ATPF0B, atpF*	ATP synthase subunit B	/	/	-1.051844	-1.370558	-1.783724	-1.76669	-1.577814	-1.391811
orf2057	*ATPF1D, atpH*	F0F1 ATP synthase subunit delta	/	/	/	-1.269876	-1.615655	-1.667103	-1.594624	-1.302267
orf2058	*ATPF1A, atpA*	ATP synthase subunit alpha	/	/	/	-1.088211	-1.50464	-1.575348	-1.511965	-1.139905
orf2059	*ATPF1G, atpG*	F0F1 ATP synthase subunit gamma	/	/	/	-1.12453	-1.616107	-1.632777	-1.367676	-1.26374
orf2091	*petF*	Ferredoxin	/	/	/	/	/	-1.019241	-1.253178	/
orf2121	*petA*	Apocytochrome f	/	/	/	/	-1.275579	-1.564538	-1.404608	/
orf2122	*petC*	Cytochrome b6-f complex iron-sulfur subunit	/	/	/	/	-1.462803	-1.577591	-1.370307	/
orf2306	*psaD*	Photosystem I reaction center subunit II	/	/	-1.255751	-1.586505	-2.30004	-2.302488	-2.049052	-1.709965
orf2316	*psb27*	Photosystem II protein Psb27	/	/	/	-1.072767	-1.663375	-1.951865	-1.773887	-1.405001
orf2448	*psb28-2*	Photosystem II reaction center protein Psb28	/	/	/	-1.024929	-1.577191	-1.296547	/	-1.296862
orf2456	*apcC*	Phycobilisome core linker protein	/	/	/	/	/	-1.380081	-1.352839	-1.021501
orf2457	*apcB*	Allophycocyanin subunit beta	/	-1.024325	-1.208384	-1.675588	-2.316682	-2.434112	-2.461914	-2.025557
orf2458	*apcA*	Allophycocyanin subunit alpha	/	/	-1.207004	-1.684449	-2.3219	-2.454783	-2.396376	-2.061688
orf2517	*psbH*	Photosystem II reaction center protein H	/	/	/	/	-1.180627	-1.141909	/	/
orf2537	*psb28*	Photosystem II reaction center protein Psb28	/	/	/	/	-1.111062	-1.03049	/	/
orf2689	*apcF*	Phycobiliprotein beta chain	/	/	/	-1.070592	-1.957198	-2.001398	-1.943838	-1.732666
orf2950	*petF*	Ferredoxin	/	/	/	/	-1.116738	/	/	/
orf3286	*psbU*	Photosystem II extrinsic protein	/	/	/	/	-1.487493	-1.471319	-1.328205	-1.047037
orf3379	*psaC*	Photosystem I subunit VII	/	/	/	-1.006419	-2.123557	-1.898789	-2.059773	-1.751667
orf3634	*psbX*	Photosystem II PsbX protein	/	/	/	/	-1.442145	-1.276505	-1.001694	-1.217276
**Porphyrin and chlorophyll Metabolism**										
orf221	*chlH, bchH*	Magnesium chelatase subunit H	/	/	/	-1.160131	-1.911091	-2.130854	-1.795053	-1.619046
orf304	*hemE, UROD*	Uroporphyrinogen decarboxylase	/	/	/	/	-1.947086	-1.936584	-1.427503	-1.431571

orf962	*HO, pbsA1, hmuO*	Heme oxygenase (biliverdin-producing)	/	/	/	-1.308417	-2.2459	-2.324744	-2.089241	-2.103153
orf1136	*acsF, chlE*	Magnesium-protoporphyrin IX monomethyl ester (oxidative) cyclase	/	/	/	/	-1.730521	-1.624501	-1.293144	-2.320904
orf1528	*chlL*	Ferredoxin:protochlorophyllide reductase (ATP-dependent) iron-sulfur ATP-binding protein	/	/	-1.021894	-1.326652	-2.117079	-2.412089	-1.90514	-1.802095
orf1530	*chlN*	Ferredoxin:protochlorophyllide reductase (ATP-dependent) subunit N	/	/	/	/	-1.875984	-2.310835	-1.525526	-1.542646
orf1769	*hemA*	Glutamyl-tRNA reductase	/	/	/	-1.167893	-1.889357	-1.628732	-1.535361	-1.157239
orf1801	*cobH-cbiC*	Precorrin-8X methylmutase	-1.102074	/	/	/	/	/	/	/
orf1802	*cobI-cbiL*	Precorrin-2 C(20)-methyltransferase	-1.243997	/	/	/	/	/	/	/
orf1998	*acsF, chlE*	Magnesium-protoporphyrin IX monomethyl ester (oxidative) cyclase 1	/	/	/	-1.312134	-2.425128	-2.42925	-1.916258	-1.662659
orf2087	*hemC, HMBS*	Hydroxymethylbilane synthase	/	/	/	/	-1.020639	-1.3712	-1.245735	-1.083423
orf2157	*chlP, bchP*	Geranylgeranyl reductase	/	/	/	/	-2.19933	-2.420374	-2.076469	-1.749383
orf2339	*COX10, ctaB, cyoE*	Protoheme IX farnesyltransferase	/	/	/	/	/	/	/	-1.123575
orf2340	*COX15, ctaA*	Heme A synthase	/	/	/	/	-1.163557	/	-1.003792	-1.367286
orf2571	*chlB*	Ferredoxin:protochlorophyllide reductase (ATP-dependent) subunit B	/	/	/	/	-2.143678	-2.441956	-1.973517	-1.794203
orf2593	*cobB-cbiA*	Cobyrinic acid a,c-diamide synthase	/	/	/	/	-1.159765	/	/	/
orf2613	*bciB*	Hypothetical protein	/	/	/	/	-1.68647	-1.68953	-1.269966	-1.154242
orf3679	*BLVRA, bvdR*	Gfo/Idh/MocA family oxidoreductase	/	/	/	/	/	-1.07051	-1.173084	-1.254367
orf3768	*pcyA*	Phycocyanobilin:ferredoxin oxidoreductase	/	/	/	/	/	-1.041524	-1.030239	/
orf3782	*CPOX, hemF*	Coproporphyrinogen III oxidase	/	/	/	-1.176253	-1.753001	-1.967197	-1.883035	-1.767562
orf3918	*hemB, ALAD*	Delta-aminolevulinic acid dehydratase	/	/	/	/	-1.494445	-1.450056	-1.408465	-1.520044
orf4253	*EARS, gltX*	Glutamate--tRNA ligase	/	/	/	/	-1.292691	-1.709524	-1.284351	-1.200924
**Carbohydrate Metabolism**										
orf023	*ISA, treX*	Glycogen debranching enzyme	/	/	/	/	/	/	-1.201634	-1.072206
orf149	*GAPDH, gapA*	Type I glyceraldehyde-3-phosphate dehydrogenase	/	/	/	-1.060369	-1.124256	/	/	/
orf291	*PRK, prkB*	Phosphoribulokinase	/	/	/	/	/	-1.098592	/	/
orf664	*PGK, pgk*	Phosphoglycerate kinase	/	/	/	-1.1844	-1.753299	-1.988866	-1.984338	-1.611036

orf959	*tktA, tktB*	Transketolase	/	/	/	/	-1.39782	-1.126337	/	/
orf1011	*gmd, GMDS*	GDP-mannose 4,6-dehydratase	/	/	-1.072568	-1.145948	-1.006162	/	/	/
orf1085	*gap2*	Glyceraldehyde-3-phosphate dehydrogenase 2	/	/	-1.170231	-1.431752	-2.190859	-2.241147	-2.038929	-1.776055
orf1156	*nanE*	N-acetylmannosamine-6-phosphate 2-epimerase	/	-1.284792	-1.297003	-1.54504	-1.389105	-1.462729	-1.028215	/
orf1212	*PDHB, pdhB*	Alpha-ketoacid dehydrogenase subunit beta	/	/	/	-1.00962	-1.590848	-1.448146	-1.110622	/
orf1326	*glgA*	Starch synthase	/	/	-1.172735	-1.257534	-1.780259	-2.161627	-2.058277	-1.499022
orf1421	*DLD, lpd, pdhD*	Dihydrolipoyl dehydrogenase	/	/	/	/	/	/	-1.042142	/
orf1443	*treZ, glgZ*	Malto-oligosyltrehalose trehalohydrolase	/	/	/	/	-1.541588	-1.445924	-1.05268	-1.063685
orf1485	*K16149*	Glycoside hydrolase	/	/	/	/	-1.286263	-1.651033	-1.702122	-1.269093
orf1770	*glpX-SEBP*	Fructose-bisphosphatase class II	/	/	-1.254271	-1.578668	-2.147261	-2.208697	-2.169432	-1.730002
orf1864	*PK, pyk*	Pyruvate kinase	/	-1.089382	/	/	-1.056363	/	/	/
orf1870	*glgC*	Glucose-1-phosphate adenylyltransferase	/	/	-1.24163	-1.436757	-2.004339	-2.141168	-1.834495	-1.662528
orf2128	*UXS1, uxs*	NAD-dependent epimerase/dehydratase family protein	/	/	-1.044495	-1.050978	-1.351149	-1.68754	-1.511221	-1.149849
orf2319	*pgm*	Alpha-D-glucose phosphate-specific phosphoglucomutase	/	/	/	/	/	/	-1.078875	/
orf2329	*glgA*	Starch synthase	/	/	/	/	-1.068973	-1.228965	-1.238921	/
orf2519	*GBE1, glgB*	1,4-alpha-glucan branching enzyme	/	/	/	/	/	-1.091795	-1.198979	/
orf2595	*pckA*	Phosphoenolpyruvate carboxykinase (ATP)	/	/	/	/	-1.690188	-1.694098	-1.327352	-1.473663
orf3143	*rpe, RPE*	Ribulose-phosphate 3-epimerase	/	/	/	-1.070903	-1.664308	-1.837612	-1.81189	-1.481863
orf4218	*rfbE*	CDP-paratose 2-epimerase	/	/	-1.178217	-1.037836	-1.344658	/	/	/
orf4219	*rfbS*	NAD(P)-dependent oxidoreductase	/	/	-1.24379	/	-1.895303	/	/	/
orf4439	*FBA, fbaA*	Fructose-1,6-bisphosphate aldolase	/	/	/	/	/	-1.049788	-1.148942	/
**Amino acid Metabolism**										
orf2583	*psp*	Histidine phosphatase family protein	/	-1.334204	-1.297605	-1.574947	-1.842474	-1.889604	-1.713603	-1.439689
orf2776	*serA, PHGDH*	Phosphoglycerate dehydrogenase	/	-1.376746	-1.314191	-1.519293	-1.410412	/	/	/
orf4389	*trpA*	Tryptophan synthase subunit alpha	/	-1.044023	-1.127266	-1.464781	-1.401029	-1.376036	/	/
**Lipid Metabolism**										
orf260	*accC*	Acetyl-CoA carboxylase biotin carboxylase subunit		-1.053166	-1.170331	-1.472398	-1.435671	-1.186436	/	/
orf960	*fabI*	3-oxoacyl-[acyl-carrier-protein] synthase 2	/	/	/	/	/	-1.244559	-1.270107	/

orf961	*acpP*	Acyl carrier protein	/	/	/	/	-1.134346	-1.445378	-1.120403	-1.00068
orf1757	*E1.14.19.3*	Acyl-CoA desaturase	/	-1.000802	-1.032509	-1.412987	/	/	/	/
orf2241	*accB, bccP*	Acetyl-CoA carboxylase biotin carboxyl carrier protein subunit	/	-1.22623	-1.160208	-1.553536	-1.402846	-1.462059	-1.445981	-1.310124
orf2654	*fabA*	3-hydroxyacyl-[acyl-carrier protein] dehydratase / trans-2-decenoyl-[acyl-carrier protein] isomerase	/	/	/	/	-1.870018	-1.406499	-1.075531	/
orf2657	*fabD*	Polyketide synthase	/	/	/	/	-1.281834	/	/	/
orf2735	*fabI*	Enoyl-[acyl-carrier protein] reductase I	/	/	/	/	-1.299971	-1.286843	-1.219711	/
orf2818	*fabZ*	Beta-hydroxyacyl-ACP dehydratase	/	-1.011676	-1.223857	-1.290788	-1.21654	-1.243417	-1.023701	-1.265194
orf3348	*fadD*	Long-chain acyl-CoA synthetase	/	/	/	/	/	-1.02831	-1.247851	-1.116828
orf3378	*ACSS, acs*	Acetyl-coenzyme A synthetase	/	-1.027548	-1.166988	-1.178968	-1.388212	-1.396544	-1.451172	-1.228425
**Carbon fixation**										
orf3712	*ccmK*	Microcompartments protein	/	/	-1.084617	-1.544011	-2.013013	-2.332908	-1.993407	-1.895019
orf3713	*ccmK*	Carbon dioxide-concentrating mechanism protein CcmK	/	/	/	/	-1.531553	-1.598937	-1.74274	-1.329245
orf3714	*ccmL*	Carbon dioxide concentrating mechanism protein CcmL	/	/	-1.321839	-1.722478	-2.074549	-2.318592	-1.875113	-1.806591
orf3715	*ccmM*	Carbon dioxide concentrating mechanism protein CcmM	/	/	-1.146145	-1.547201	-2.015334	-2.072119	-1.934726	-1.735068
orf3716	*ccmN*	Carbon dioxide concentrating mechanism protein	/	-1.014512	-1.299227	-1.659696	-2.017927	-2.022599	-1.706354	-1.596323
orf3717	*rbcL*	Ribulose-bisphosphate carboxylase large subunit	/	/	-1.072348	-1.386194	-2.227396	-2.262108	-1.991006	-1.767632
orf3719	*rbcS*	Ribulose bisphosphate carboxylase small subunit	/	/	-1.274913	-1.460708	-2.125579	-2.269645	-1.779566	-1.747609
**Oxidative phosphorylation**										
orf945	*ndhD*	NAD(P)H-quinone oxidoreductase subunit D4	/	/	/	/	-1.573704	-1.778697	-1.564252	-1.216357
orf947	*ndhF*	NAD(P)H-quinone oxidoreductase subunit F	/	/	/	/	-1.349375	-1.416703	-1.564401	-1.152541
orf1155	*ppa*	Inorganic pyrophosphatase	/	/	/	-1.40926	-1.678185	-1.708454	-1.733365	-1.328974
orf1234	*ndhA*	NADH-quinone oxidoreductase subunit H	/	/	/	/	-1.073315	-1.133462	-1.296859	-1.01551
orf1235	*ndhI*	NAD(P)H-quinone oxidoreductase subunit I	/	/	/	/	-1.199853	-1.0175	/	/
orf1236	*ndhG*	NADH:ubiquinone oxidoreductase subunit J	/	/	/	/	-1.08541	/	/	/
orf1402	*ATPF1B, atpD*	ATP synthase subunit beta	/	/	/	-1.259526	-1.859781	-2.045115	-2.025824	-1.671682
orf1403	*ATPF1E, atpC*	ATP synthase epsilon chain	/	/	-1.019071	-1.265868	-1.879405	-1.999593	-1.650046	-1.492712

orf1818	*ndhM*	NAD(P)H-quinone oxidoreductase subunit M	/	/	-1.136791	-1.122891	-1.956112	-1.416938	/	-1.185531
orf2053	*ATPF0A, atpB*	AtpB	/	/	/	/	-1.496058	-1.67446	-1.660346	-1.270855
orf2054	*ATPF0C, atpE*	ATP synthase F0 subunit C	/	/	/	-1.03497	-1.413663	-1.793516	-2.041615	-1.421271
orf2055	*ATPF0B, atpF*	F0F1 ATP synthase subunit B'	/	/	-1.269863	-1.508116	-1.720013	-1.840708	-1.617089	-1.458756
orf2056	*ATPF0B, atpF*	ATP synthase subunit B	/	/	-1.051844	-1.370558	-1.783724	-1.76669	-1.577814	-1.391811
orf2057	*ATPF1D, atpH*	F0F1 ATP synthase subunit delta	/	/	/	-1.269876	-1.615655	-1.667103	-1.594624	-1.302267
orf2058	*ATPF1A, atpA*	ATP synthase subunit alpha	/	/	/	-1.088211	-1.50464	-1.575348	-1.511965	-1.139905
orf2059	*ATPF1G, atpG*	F0F1 ATP synthase subunit gamma	/	/	/	-1.12453	-1.616107	-1.632777	-1.367676	-1.26374
orf2249	*ndhF*	NAD(P)H-quinone oxidoreductase subunit F	/	/	/	/	/	-1.401208	-1.301029	-1.320056
orf2339	*COX10, ctaB, cyoE*	Protoheme IX farnesyltransferase	/	/	/	/	/	/	/	-1.123575
orf2340	*COX15, ctaA*	Heme A synthase	/	/	/	/	-1.163557	/	-1.003792	-1.367286
orf2341	*coxB, ctaC*	Cytochrome C oxidase subunit II	/	/	/	/	-1.485754	-1.686257	-1.465255	-1.202403
orf2342	*coxA, ctaD*	Cytochrome c oxidase subunit I	/	/	/	/	-1.49678	-1.698612	-1.479239	-1.185729
orf2343	*coxC, ctaE*	Heme-copper oxidase subunit III	/	/	/	/	-1.399835	-1.50053	-1.269476	-1.03879
orf2351	*sdhC, frdC*	Putative succinate dehydrogenase cytochrome b subunit	/	/	/	/	-1.020112	-1.152493	-1.202111	-1.055951
orf3477	*ndhD*	NAD(P)H-quinone oxidoreductase subunit D4	/	/	/	-1.156445	-2.079242	-2.204658	-2.075789	-1.788747
orf4060	*ndhB*	NAD(P)H-quinone oxidoreductase subunit 2	/	/	/	/	-1.06573	-1.440463	-1.560607	-1.24706
orf4254	*ndhN*	NAD(P)H-quinone oxidoreductase subunit N	/	/	/	/	-1.468605	-1.621216	-1.78127	-1.155769

TABLE S6 Transcriptional levels of copy number variation genes in the *Microcystis* genome after Mic1 infection.

Locus tag	Length (bp)	Overlap (bp)	Strand	Annotation	Log_2 FoldChange							
					0.5 h	1 h	1.5 h	2 h	4 h	6 h	8 h	10 h
orf3029	482	46	-	Hypothetical protein	/	-1.318264	-1.332001	-1.500621	-1.50249	-1.42433	-1.11091	-1.244936
orf3030	1286	1287	-	DEDD_Tnp_IS110; Transposase	/	/	/	/	/	3.502497	3.619751	3.833235
orf3031	380	279	-	Hypothetical protein	/	/	/	1.303704	/	/	/	/
orf3032	260	261	-	Hypothetical protein	/	/	/	/	/	/	/	/
orf3033	314	315	-	Hypothetical protein	/	/	/	1.498289	/	/	/	/
orf3034	1052	1053	-	Lambda repressor-like; SpoIIID	/	/	/	/	/	/	/	/
orf3035	248	249	+	Lambda repressor-like; HTH_3_3	/	/	/	/	/	1.288747	1.200416	/
orf3036	932	933	+	Lipid binding protein	/	/	/	/	1.022143	/	/	/
orf3037	236	237	+	Hypothetical protein	/	/	/	/	/	/	/	/
orf3038	569	570	-	Phage integrase	/	/	/	/	/	/	/	/
orf3039	1193	1194	+	Enolase	/	/	/	/	/	/	/	/
orf3040	2003	2004	+	Hypothetical protein	/	/	/	/	/	/	/	/
orf3041	812	813	+	Hypothetical protein	/	/	/	/	/	4.186136	4.419668	4.359621
orf3042	371	372	+	Hypothetical protein	/	/	/	/	/	/	/	/
orf3043	386	387	+	Hypothetical protein	/	/	/	/	/	4.409537	3.645612	3.962079
orf3044	401	402	+	Hypothetical protein	/	/	/	/	/	4.465152	4.391242	4.253675
orf3045	467	468	+	Hypothetical protein	/	/	/	/	/	4.172835	4.591515	5.614151
orf3046	617	618	+	N-terminal Ig-like domain in baseplate protein	/	/	/	/	/	4.101424	3.172164	5.606252
orf3047	434	435	+	Hypothetical protein	/	/	/	/	/	3.212233	/	2.037753
orf3048	800	801	+	LamG-like jellyroll fold domain-containing protein	/	/	/	/	/	2.975675	3.864765	3.607104
orf3049	230	231	+	Hypothetical protein	/	/	/	/	/	/	3.068401	3.182221
orf3050	440	441	+	D-Ala-D-Ala carboxypeptidase family metallohydrolase	/	/	/	/	1.074036	1.042993	/	1.327406
orf3051	248	57	-	Acetyltransferase (GNAT) domain	/	/	/	/	/	/	/	/
